# Mapping to Quality of Life and Capability Measures in Cataract Surgery Patients: From Cat-PROM5 to EQ-5D-3L, EQ-5D-5L, and ICECAP-O Using Mixture Modelling

**DOI:** 10.1177/2381468320915447

**Published:** 2020-04-06

**Authors:** Padraig Dixon, William Hollingworth, John Sparrow

**Affiliations:** Population Health Sciences, Bristol Medical School, University of Bristol, Bristol, UK; MRC Integrative Epidemiology Unit, Bristol Medical School, University of Bristol, Bristol, UK; Population Health Sciences, Bristol Medical School, University of Bristol, Bristol, UK; Population Health Sciences, Bristol Medical School, University of Bristol, Bristol, UK; Bristol Eye Hospital, Bristol, UK

**Keywords:** Cat-PROM5 mixture models, cataract, EQ-5D, ICECAP, mapping, quality of life

## Abstract

**Objectives.** Cataract is a prevalent and potentially blinding eye condition. Cataract surgery, the only proven treatment for this condition, is a very frequently undertaken procedure. The objective of this analysis was to develop a mapping algorithm that could be used to predict quality of life and capability scores from the Cat-PROM5, a newly developed, validated patient-reported outcome measure for patients undergoing cataract surgery. **Methods.** We estimated linear models and adjusted limited dependent variable mixture models. Data were taken from the Predict-CAT cohort of up to 1181 patients undergoing cataract surgery at two sites in England. The Cat-PROM5 was mapped to two quality of life measures (EQ-5D-3L and EQ-5D-5L) and one capability measure (ICECAP-O). All patients reported ICECAP-O and one or other of the EQ-5D measures both before and after cataract surgery. Model performance was assessed using likelihood statistics, graphical inspections of model fit, and error measurements. **Results.** Adjusted limited dependent variable mixture models dominated linear models on all performance criteria. Mixture models offered very good fit. Three component models that allowed component membership to be a function of covariates (age, sex, and diabetic status depending on specification and outcome measure) and which conditioned on covariates offered the best performance in almost all cases. An exception was the EQ-5D-5L post-surgery for which a two-component model was selected. **Conclusions.** Mapping from Cat-PROM5 to quality of life and capability measures using adjusted limited dependent variable mixture models is feasible, and the estimates can be used to support cost-effectiveness analysis in relation to cataract care. Mixture models performed strongly for both quality of life outcomes and capability outcomes.

## Introduction

Cataract, the clouding of the lens of the eye, is a prevalent and potentially blinding age-related condition. Cataract surgery is the only proven treatment for the condition and is the most frequently undertaken surgical procedure on the National Health Service (NHS)^1^ and in many other international health systems.^2^ Almost 400,000 cataract surgeries are performed annually in England.^3^ The aim of this study was to map scores from a newly created self-report questionnaire for cataracts (the Cat-PROM5—cataract patient-reported outcome measure^2^) to generic, preference-based quality of life (Euroqol EQ-5D questionnaire^4^ in both the three-level [EQ-5D-3L^5^] and five-level [EQ-5D-5L^6^] versions) and capability (ICEpop CAPability measure for Older people questionnaire^7^ [ICECAP-O]) measures.

Mapping is the expression of values from one questionnaire, scale, or instrument in terms of another. Mapping can be used to facilitate analyses that may require estimates of generic utility and capability scores in settings where (in this case) only the Cat-PROM5 instrument was used. Clinical studies may wish to avoid imposing a burden on patients to complete both a disease-specific questionnaire such as Cat-PROM5 and generic measures. There may be administrative or financial costs associated with administering multiple instruments that dispose researchers to limit the volume of data collected. These considerations are particularly relevant to cataract surgery—given the exceptionally high volume of this surgical procedure, the cost and burden of administering more than one questionnaire before and after surgery is likely to be prohibitive in most circumstances.

A growing interest in mapping is reflected in the increasing volume of applied analyses,^8–10^ methodological work,^10^ guideline development,^11,12^ and reliance on mapping in health technology appraisal submissions to the National Institute for Health and Care Excellence.^13,14^

## Data

### The Predict-CAT study

Predict-CAT was a prospective cohort study, the key objective of which was the quantification of risk indicators for favorable and unfavorable outcomes in typical National Health Service (NHS) patients who were candidates for cataract surgery. Potential participants in the cohort were identified at the time of listing for cataract surgery, or from preoperative assessment patient lists.^2^ The study was undertaken at two NHS sites in the south of England: Bristol Eye Hospital and Gloucestershire Hospitals NHS Foundation Trust. The patient pathway at both trusts was managed as for routine cataract care.

Patients willing to participate in the study were eligible for inclusion if they were aged at least 50 years at recruitment, were approaching first or second eye cataract surgery, were capable of providing informed consent, were willing to participate, and could understand and complete versions of the Cat-PROM questionnaire, versions of the Euroqol EQ-5D questionnaire,^4^ and the ICECAP-O (ICEpop CAPability measure for Older people) questionnaire.^7^ These questionnaires are described in more detail below. Patients who failed to meet entry criteria, and/or who were undergoing certain types of prespecified eye operation (such as combined phako-trabeculectomy), were not eligible for inclusion.

### Data Collection, and the Cat-PROM5 Instrument

Data from the Predict-CAT study cohort was used as the sole source of the estimation sample. Participant data were obtained on two occasions: preoperatively for a baseline assessment and postoperatively for a follow-up assessment.

At baseline, height, weight and diabetic status were assessed, and participants reported other health, demographic, and socioeconomic data. All participants completed Cat-PROM5 and capability (ICECAP-O) questionnaires and were randomized (1:1:1) to receive one of three quality of life questionnaires: either EQ-5D-3L,^5^ EQ-5D-3L with vision “bolt-on,”^10^ or EQ-5D-5L.^6^ We did not map to the EQ-5D-3L with vision “bolt-on” questionnaire due to the infrequent use of that questionnaire and unclear relevance to the calculation of quality-adjusted life years. A full ocular examination and slit lamp assessment was undertaken on all participants.

Follow-ups were scheduled to take place 6 to 8 weeks after cataract surgery, although there was some variation in when these actually took place. On confirmation of continuing eligibility, participants described any changes in clinical details, underwent a full ocular examination, and again completed the Cat-PROM5 and quality of life questionnaires administered at baseline.

### The Cat-PROM5 Instrument

The CatPROM-5 instrument was designed as a short questionnaire to measure the self-reported impact of cataracts on vision and quality of life.^2^ It has just five items: whether vision overall has been affected by the “bad” eye, the extent to which eyesight has interfered with life in general, a rating of vision overall, the frequency with which vision prevented usual activities, and difficulties in reading normal print in books or newspapers. The recall period for all questions is “the past month.” The instrument is responsive and good psychometric properties have been demonstrated.^2^

### The EQ-5D-3L and EQ-5D-5L Measures

The EQ-5D measure is a standardized, preference-based, generic questionnaire that facilitates the measurement and valuation of health-related quality of life. The EQ-5D measures five dimensions of health-related quality of life: mobility, self-care, capacity to undertake usual activities, pain and discomfort, and anxiety and depression.

The EQ-5D-3L^4^ instrument measures three categories of response to questions on each of these dimensions. The responses may be described as follows: no problems, some problems, and extreme problems. The EQ-5D-5L^6^ instrument encompasses the same five domains, but allows respondents to report five rather than three categories of response: no problems, slight problems, moderate problems, severe problems, and extreme problems. Responses to EQ-5D questionnaires can be converted into a single index utility score that is anchored on 1 for perfect health and 0 for death. Negative values represent health profiles considered to be worse than death, to a minimum under the English valuation set of −0.594 for EQ-5D-3L^5^ and −0.285 for EQ-5D-5L.^6^

### The ICECAP-O Questionnaire

The ICECAP-O questionnaire measures “capability” in older people. Capability is intended to reflect a broader sense of well-being than may be captured by the notion of “health” alone. The instrument has five attributes: attachment, security, role, enjoyment, and control. Each attribute has four levels. For example, the control attribute is intended to reflect independence, and its statements range from “I am able to be completely independent” to “I am unable to be at all independent.” Valuations for the questionnaire were obtained from a best-worst scaling exercise conducted among older people living in England.^15^ The index value has a theoretical range from 0 (lowest possible value reflecting low capability) to 1, reflecting high capability.

## Methods for Quantitative Analysis

### Best Practice in Methods for Mapping Analyses

Our objective was to map responses from the Cat-PROM5 instrument to EQ-5D-3L, EQ-5D-5L, and ICECAP-O questionnaires. Our approach to the selection of specific methods was guided by recommendations^12,16^ in best practice for mapping set out in an expert consensus report on mapping. We also report our methods and findings following the “preferred items” checklist of Petrou et al.,^17^ a completed version of which is available in the supplementary material. We consider recommendations for model selection below under specific headings.

### Empirical Performance of Methods for Mapping Analysis

Recent evidence^18–26^ suggests that a class of direct mapping algorithms that uses adjusted limited dependent variable mixture modelling offers advantages over other types of mapping methods, particularly when utility distributions are skewed and multimodal. We discuss these methods in more detail below, following further consideration of the Wailoo et al. criteria^12,16^ for best practice in mapping. We note in passing that indirect mapping could not be undertaken for the Predict-CAT cohort given zero or limited responses to some categories of both EQ-5D-3L and EQ-5D-5L

### Good Statistical Practice in Mapping

A critical rationale for mapping functions is to accurately predict, in a variety of datasets, health state utility values of the target instrument.^27^ The accuracy of predictions can be understood, in broad terms, as a measure of the “fit” between the model’s predicted utility values and the utility values reported by respondents. We relied on a variety of criteria as follows.

Summary measures of fit, such as the root mean squared error (RMSE), mean absolute error (MAE), and ranges of predictions are frequently reported in mapping analyses^27^ and offer helpful but partial and potentially insensitive characterizations of model fit.^12^ Information criteria that tradeoff between model fit and parsimony by penalizing the inclusion of additional covariates offer a means of choosing between likelihood-based models. We calculated for each model the Akaike Information Criterion (AIC) and the Bayesian Information Criterion (BIC); lower values for each criterion are preferred to higher values.

We also used less formal but potentially informative tools such as assessing face validity and graphical comparisons of plots of actual and predicted quality of life and capability values, and comparisons of actual and predicted values across the distribution of observed values.

### Covariate Selection

Two important but potentially conflicting issues are relevant to the a priori choice of the covariate set.^12^ The first is that the exclusion of covariates risks misspecification of the mapping model, subject to the proviso that correlated covariates need not be included in the interests of parsimony. The second issue is that of overfitting, an important consideration given the relatively modest size of our cohort.

Age and sex are associated with quality of life in general,^28–30^ and with the Cat-PROM5 instrument in the Predict-CAT cohort. Age and sex are correlated with other available covariates, are likely to be available in most other datasets to which the mapping algorithm developed here may be applied, and are frequently used in mapping algorithms of the type developed here. Nonlinear terms in age did not improve the predictive performance of any studied model in initial modelling. Diabetes is associated with decrements to utility in general^29,30^ and in this cohort (e.g., diabetic participants had baseline mean EQ-5D-3L values about 0.06 points lower than non-diabetic participants), and is a risk factor for cataract.^31,32^ Age, sex, and diabetic status were therefore retained as covariates.

### External Validation of Algorithms

**Figure 1:**
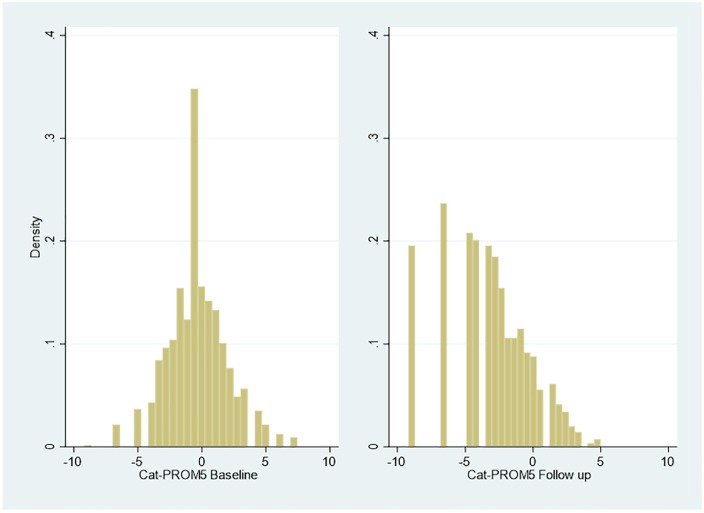
Responses to Cat-PROM5 at baseline and follow-up.

**Figure 2:**
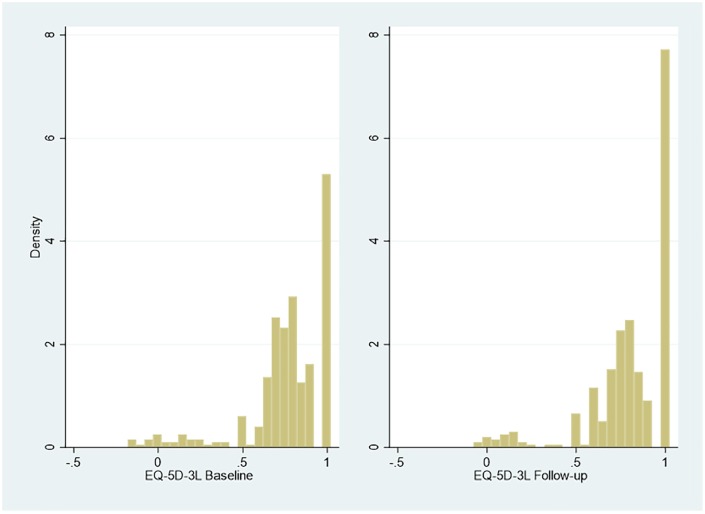
Responses to EQ-5D-3L at baseline and follow-up.

**Figure 3:**
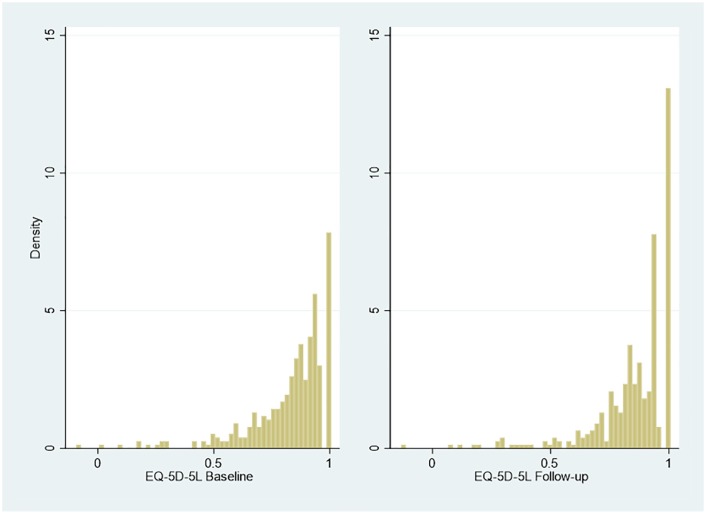
Responses to EQ-5D-5L at baseline and follow-up.

**Figure 4:**
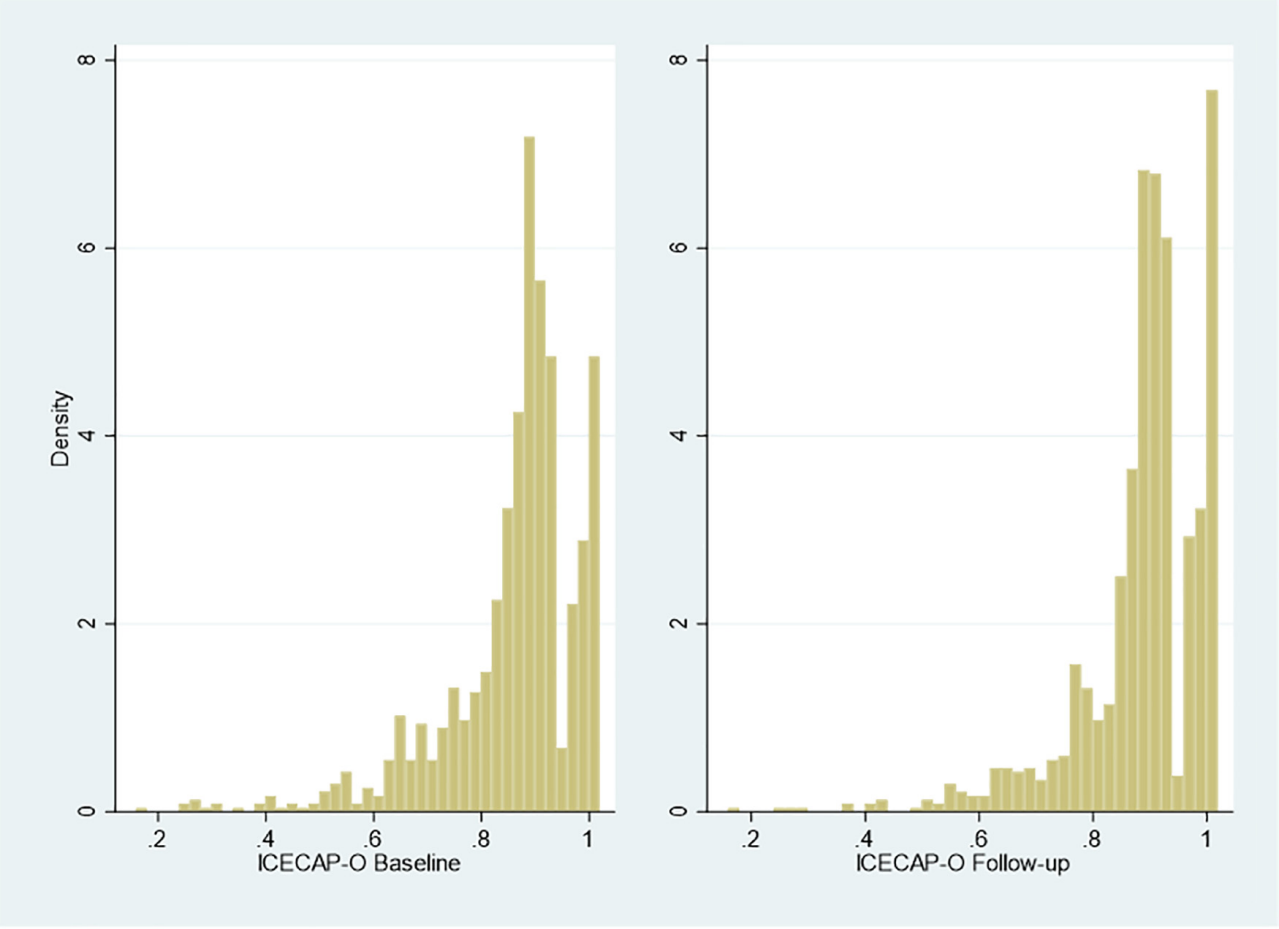
Responses to ICECAP-O at baseline and follow-up.

No other sources of data were available given the ab initio creation of the Cat-PROM5 instrument during an earlier phase of this research program. The pre- and postscores were not good candidates for validation as the distributions of utility and capability scores were substantially different in the pre- and postoperative settings (see in particular Figures 1 to 4). This means that a mapping algorithm that worked well in the preoperative setting may not have been well suited to the postoperative context because of differences in the distributions in both the source and target instruments. To avoid imposing a single mapping algorithm across these distinct circumstances, we instead rely on validation by head-to-head performance comparisons between types of estimators and model specifications.^12^

### Exploratory Data Analysis and Missing Data

We undertook exploratory data analysis by calculating Spearman’s correlation coefficient, calculating summary statistics, comparing ranges, and calculating EQ-5D and ICECAP-O measures at different levels of the Cat-PROM5 instrument. We retained individuals in the analysis sample if they had complete data at both baseline and follow-up.

### Models Estimated

We estimated two classes of mapping algorithms. The first set of models used an adjusted limited dependent variable, mixture modelling approach.^18^ We compared these findings to simple linear models estimated using ordinary least squares (OLS). Linear models estimated using OLS were studied for two reasons. The first was to provide a benchmark against which more complex models could be compared. The second was to assess whether this estimator was capable of outperforming these other, more complex models.

The use of mixtures of distributions readily permits their range to be limited to reflect the minimum and maximum permitted values at feasible values of the UK or English tariff for each of the quality of life and capability questionnaires studied here. The distributions can also model the “gap” between perfect health (EQ-5D-3L state 11111, index value = 1) and the next highest index value in the EQ-5D distributions (“some problems” with usual activities, “no problems” on other dimensions EQ-5D-3L state 11211, index value = 0.883). This is larger than any other discontinuity in the UK valuation of EQ-5D-3L^18^ and may reflect the large perceived difference in utility between the labels “no problems” and “some problems.” The discontinuity is still present, but much smaller on the EQ-5D-5L (EQ-5D-5L 11111, index value = 1; EQ-5D-5L 11211, index value = 0.951), which has more response levels and uses the label “slight problems” to define the first level of decrement in usual activities.

Membership of latent classes, which may give rise to the familiar multimodal “peaks” in target distributions, is given effect under the mixture modelling approach by using multinomial logit models (for the probability of latent class membership) that can include variables that may affect the probability of class membership such as age or sex. These latent classes were combined using probability weights to generate a skewed and multimodal distribution. There is no simple rule to determine how many classes ought to be used, and a degree of judgement is required.^18^

We use finite mixtures of limited dependent variable normal distributions to model the distributions of latent classes. Beta-type models are an alternative,^33^ but are computationally more expensive, require more parameters than mixtures of normal distributions, and were not obviously well suited to the relatively modest sample sizes available to us.

### Approach to Model Development

The dependent variable in all regressions is an EQ-5D index score (whether the EQ-5D-3L score or the EQ-5D-5L score) or the ICECAP-O index score. All models included the Cat-PROM5 summary index value. Model fit and model convergence was not improved when including the components of the Cat-PROM5 index in initial modelling.

Mixture models with one component were dominated by mixture models with more than one component. Mixture models with four components almost never converged. We therefore focused attention in all subsequent modelling on two- or three-class component models. All EQ-5D models explicitly incorporated the gap between perfect health and the next highest possible value.

The results of mixture models can be sensitive to model starting values. We tested several approaches to the choice of starting values, given the sensitivity of mixture models to these parameters. The approach adopted was to first estimate a constant-only model, the parameters of which were then used as starting values for full models. The exception was when modelling baseline EQ-5D-3L using three components, for which the most stable estimates were obtained by a search for starting values using Stata’s inbuilt maximum likelihood algorithm. No approach to starting values was found to produce convergent values for the EQ-5D-3L outcome at follow-up.

Following these initial analyses, we explored mixture models of increasing complexity. We started with simple models with no covariates other than the Cat-PROM5 index score, and with fixed probabilities of component membership. We then progressively extended these models to include all single and joint combinations of sex, age, and diabetic status as covariates, and as variables singly and jointly influencing the probability of component membership. For linear models, we initially considered models with no covariates other than the Cat-PROM5 index value, before estimating models including all combinations of sex, age, and diabetic status as covariates.

All analysis was conducted using Stata version 15.1 (StataCorp: College Station, Texas). The mixture modelling approach was implemented with the *-aldvmm-* package.^21^

## Results

Complete data at both baseline and follow-up appointments were available from 1181 different participants of whom 598 were women (51%). However, complete data (both baseline and follow-up assessments) on target outcome measures (both EQ-5D measures and ICECAP-O) were not available for all of these individuals. Mean age at baseline was 73.8 years (standard deviation: 8.2). There were 226 (19%) diabetic participants at baseline.

Mean quality of life and capability were higher at follow-up than at baseline, and the proportion of participants reporting “best” scores on all measures increased (Table 1).

**Table 1:** Summary Statistics

	Baseline				Follow-Up			
	EQ-5D-3L (n = 396)	EQ-5D-5L (n = 383)	ICECAP-O (n = 1174)	CatPROM5 (n = 1181)	EQ-5D-3L (n = 396)	EQ-5D-5L (n = 383)	ICECAP-O (n = 1174)	CatPROM5 (n = 1181)
Mean	0.76	0.83	0.86	−0.31	0.80	0.85	0.89	−3.20
SD	0.24	0.17	0.12	2.34	0.23	0.17	0.11	3.08
Minimum	−0.18	−0.1	0.16	−9.18	−0.08	−0.13	0.16	−9.18
Maximum	1.00	1.00	1.00	7.45	1.00	1.00	1.00	4.98
% of “best” values	26.5%	15.7%	9.7%	0.1%	38.6%	26.4%	15.4%	9.2%

All quality of life and capability index values were correlated with the Cat-PROM5 instrument with the expected sign (Table 2)—better outcomes are associated with a negative score on Cat-PROM5. *P* values were <0.001 for all questionnaires at both time points.

**Table 2:** Correlation Between Quality of Life/Capability and Cat-PROM5

	Baseline			Follow-Up		
	EQ-5D-3L	EQ-5D-5L	ICECAP-O	EQ-5D-3L	EQ-5D-5L	ICECAP-O
Spearman’s rho	−0.20	−0.30	−0.35	−0.20	−0.26	−0.29

### Distributions of Source and Target Instruments

Figure 1 summarizes the distribution of Cat-PROM5 at baseline and follow-up (n = 1186 at each time point).

This indicates an improvement in overall cataract-related outcomes, confirming the improvements reported in Table 1, with a leftward shift in the index value of Cat-PROM5 apparent at follow-up compared to baseline. Figures 2 and 3 summarize baseline and follow-up EQ-5D-3L (n = 396) and EQ-5D-5L (n = 383) index utilities.

Multimodality and gaps that are the classic features of EQ-5D distributions.^10,18^ The distribution of the ICECAP-O index values (n = 1174) is similar (Figure 4).

### Performance of Mapping Models

OLS models failed to reproduce many features of the target distributions. For example, the linear model with the lowest mean square error (0.231) for EQ-5D-3L at baseline had a higher MSE than that reported in Table 3 for mixture models applied to this outcome (0.229), and likewise had a higher mean average error (0.161 compared with 0.160 in the mixture models). Of more consequence is that the lowest predicted value of EQ-5D-3L value was 0.497 (compared with an observed minimum of −0.181) and a maximum of 0.995 compared with 1 in the observed data. Moreover, these models predicted values outside the feasible permitted range; approximately 3% of values were predicted to be in the “gap” between 0.883 and 1. The distribution of predicted values was approximately symmetric, unlike the skewed and multimodal values of the observed data. The same types of issue were observed from linear models of EQ-5D-5L and ICECAP-O at baseline and follow-up.

**Table 3:** Model Performance of Selected Specifications^a^

	Baseline			Follow-Up	
	EQ-5D-3L (n = 396)	EQ-5D-5L (n = 383)	ICECAP-O (n = 1174)	EQ-5D-5L (n = 383)	ICECAP-O (n = 1174)
Specification: Covariates	Cat-PROM5, age, sex, and diabetic status	Cat-PROM5, age, sex, and diabetic status	Cat-PROM5, age, sex, and diabetic status	Cat-PROM5, age, and sex	Cat-PROM5, age, and sex
Specification: Variables influencing component membership	Cat-PROM5, sex, and diabetic status	Cat-PROM5, age, and, diabetic status	Cat-PROM5, age, and sex	Cat-PROM5, sex, and diabetic status	Cat-PROM5, sex, and diabetic status
Number of components	3	3	3	2	3
RMSE	0.229	0.154	0.106	0.161	0.104
MAE	0.160	0.111	0.073	0.112	0.072
AIC	−149.588	−296.507	−1447.194	−766.444	−1468.820
BIC	−81.904	−247.984	−1348.123	−717.198	−1391.635

AIC, Akaike information criterion; BIC, Bayesian information criterion; MAE, mean absolute error; RMSE, root mean square error.

a Convergent models could not be identified for EQ-5D-3L at follow-up.

Adjusted limited dependent variable mixture models performed better than OLS models on all criteria for both versions of EQ-5D, and for ICECAP-O. Our focus hereon is therefore on the performance of the mixture models.

No single mixture model was superior when assessed against all performance criteria. Generally, models that included some covariates and which allowed the probability of class membership to depend on covariates performed better than simpler models. Three-component models outperformed two-component models on almost all performance criteria, except for EQ-5D-5L at follow-up, for which the best performing two-component model had very slightly better error performance than the three-component model.

Inspection of graphical output revealed that most mixture models had good fit to target distributions. Models with relatively low error scores also had good face validity, with the associations between target questionnaires and Cat-PROM5 having the expected sign.

There is a risk that identifying a single model as the “winner” creates an artificial distinction between this and other models despite similarities of performance. However, given that future analysts may nevertheless require a specific set of parameter estimates to make a mapping between Cat-PROM5 and the three target questionnaires modelled here, Table 3 presents models with the lowest RMSE, provided that these models had good face validity, and that at least one other criterion (AIC, BIC, MAE) was better than the median performance (across all estimated models for the target outcome concerned) for that criterion.

The associated parameter estimates and associated covariance matrices for these five models are available as spreadsheets in supplementary material available at https://github.com/pdixon-econ/cat-prom5-mixture-mapping. Table 4 shows further selected performance metrics from these models.

**Table 4:** Comparison of Predictions to Actual Data^a^

	Baseline			Follow-Up	
	EQ-5D-3L (n = 396)	EQ-5D-5L (n = 383)	ICECAP-O (n = 1174)	EQ-5D-5L (n = 383)	ICECAP-O (n = 1174)
Predicted mean outcome	0.76	0.83	0.86	0.85	0.89
Actual mean outcome	0.76	0.83	0.86	0.85	0.89
Predicted standard deviation of outcome	0.25	0.17	0.12	0.17	0.11
Actual standard deviation of outcome	0.24	0.17	0.12	0.17	0.11
Predicted proportion in perfect health	27.2%	18.1%	10.6%	30%	16.3%
Actual proportion in perfect health	26.5%	15.7%	9.7%	26.4%	15.3%
Predicted minimum outcome	−0.53	−0.24	0.00	−0.013	0.09
Actual minimum outcome	−0.18	−0.1	0.16	−0.013	0.16

a Convergent models were not obtained for EQ-5D-3L at follow-up.

The results indicate accurate prediction of the mean and standard deviation of all outcomes. No values were reported outside feasible ranges for any of these models. There is some modest overprediction at the tails of all outcome distributions, which can also be seen when comparing predicted values from simulated data (using 1000 simulated values from the estimated mixture models) to actual data on each target outcome variable (see Figures A1 and A2 in supplementary material).

Despite this modest overprediction at the extremes of the distributions, there is a good fit between the simulated data produced by each model and the actual data. Finally, 95% confidence intervals by decile of the Cat-PROM5 overlap those of predicted values for all models—see supplementary material for a graphical summary of this output.

Overall, adjusted limited dependent variable mixture models offer a good to excellent fit. In this cohort, selected models reproduced important features of target outcome distributions. The models reflected mean values by decile of Cat-PROM5, reproduced the skewness and multimodality of target outcome distributions, and did not predict any values outside feasible ranges. Supplementary material (available at https://github.com/pdixon-econ/cat-prom5-mixture-mapping) contains Stata code, based on Gray et al.,^22^ to implement the mapping algorithm in other samples.

## Discussion

This study reports the findings of the first mapping from the new Cat-PROM5 instrument to EQ-5D-3L, EQ-5D-5L, and ICECAP-O measures. The mapping algorithms will be relevant to clinical and research settings that involve cataracts, one of the most prevalent eye conditions in the world. The algorithms may be used on preoperative and postoperative patients. Parameter estimates and covariance matrices are available in supplementary material (https://github.com/pdixon-econ/cat-prom5-mixture-mapping) to allow other researchers to use these estimates.

This is also, to our knowledge, the first set of mapping algorithms to use adjusted limited dependent variable mixture models with ICECAP-O as an outcome, and one of the first studies to map from a disease specific measure to ICECAP-O.^34^ The results and methodology may also be relevant to mapping studies in other disease areas where patients report substantial differences in preference-based outcomes before and after clinical interventions. This may include, for example, before-and-after outcome assessments in relation to joint replacements.

We note at this juncture that the development of a successful mapping algorithm for Cat-PROM5 does not mean that the target preference-based genetic instruments we studied are necessarily the most appropriate means of measuring outcomes in patients undergoing cataract surgery. This kind of assessment is beyond the scope of our work. However, the mapping algorithms will permit the calculation of quality-adjusted life years and other measures intended to offer comparable information on effectiveness and cost-effectiveness across different types of intervention, health conditions, and patient groups. These outcome measures are fundamental to health technology appraisal and reimbursement decisions, and the mapping algorithm extends the domains in which the Cat-PROM5 instrument may be used.

### Strengths

We reviewed guidelines for conducting^12^ and reporting^11^ mapping studies. Where possible and appropriate, we sought to adhere to these guidelines in the analysis and presentation of our models. We paid particular attention to the selection of modelling approaches that had been demonstrated to perform well in other disease areas and for other disease-specific instruments.

To this end, we focused on adjusted dependent variable limited dependent mixture models. These models offer a flexible basis for developing mapping algorithms. Mapping using adjusted limited dependent variable mixture models offered excellent fit to each target outcome measure. The mixture model approach offers much more accurate prediction across the distribution of the target outcomes than linear models, which will be relevant in future modelling exercises where a range of possible health states may be included in, for example, decision-analytic models used for cost-effectiveness analysis.

### Limitations

Differences in the distributions of each measure between baseline and follow-up meant that using one or other of these samples as a validation sample would be inappropriate, since the model specifications chosen at baseline was not likely to be suitable for the follow-up data. For example, the proportion of patients in perfect health increased by more than 50% for EQ-5D-5L between baseline and follow-up.

While validation on external samples is not necessarily required by ISPOR (International Society for Pharmacoeconomics and Outcomes Research) guidelines,^12^ there would be merit in exploring the robustness of these models on other, larger datasets that may become available in the future. In the absence of data from larger external studies, we suggest that those interested in mapping from Cat-PROM5 to the target outcome measures distinguish between preoperative and postoperative cataract patients for the reasons described above.

The analysis involved fitting over 900 different models, excluding preliminary data investigations and modelling. No single model was superior on all assessment criteria, and many of the better performing models were practically indistinguishable when assessing graphical outputs. It is possible that, by emphasizing RMSE as the principal means of discriminating between the many mixture models that had good fit, we have overlooked other models that may be more suitable in other patient cohorts.

More generally, it is not possible to know if the estimation models are misspecified. There is a tradeoff between including many potentially relevant covariates and allowing the mapping algorithm to be general enough for use in context where less rich patient-level data are available. However, it is reassuring that many mixture models that relied on individual patient data (in some form) on sex, age, and diabetic status offered (at a minimum) satisfactory performance.

There is no guarantee that global rather than local optima were identified in the mixture models, despite efforts to use a variety of approaches to assess the sensitivity of results to different starting models. Convergence was not achieved for any specification of EQ-5D-3L at follow-up. This requires exploration in larger samples of cataract patients.

Between the end of data collection in the Predict-CAT cohort and the writing of this report, a quality review of the valuation process for the EQ-5D-5L questionnaire was published.^35^ This review raised a number of concerns, which led to NICE (National Institute for Health and Care Excellence) issuing a position statement recommending that this valuation not be used by organizations preparing submissions to NICE. The future of the valuation is uncertain. We note that mixture modelling between Cat-PROM5 and EQ-5D-5L using the current valuation for England offers excellent model fit, but changes to valuation would change the parameters of this mapping model.

## Conclusion

Mapping to EQ-5D-3L, EQ-5D-5L, and ICECAP-O from the newly developed Cat-PROM5 instrument for patients eligible for cataract surgery is feasible. Mapping using adjusted limited dependent variable mixture models offered good to excellent fit for preoperative and postoperative patient cohorts.

## Supplemental Material

Appendix_1_online_supp – Supplemental material for Mapping to Quality of Life and Capability Measures in Cataract Surgery Patients: From Cat-PROM5 to EQ-5D-3L, EQ-5D-5L, and ICECAP-O Using Mixture Modelling Click here for additional data file. Supplemental material, Appendix_1_online_supp for Mapping to Quality of Life and Capability Measures in Cataract Surgery Patients: From Cat-PROM5 to EQ-5D-3L, EQ-5D-5L, and ICECAP-O Using Mixture Modelling by Padraig Dixon, William Hollingworth and John Sparrow in MDM Policy & Practice

Appendix_2_online_supp – Supplemental material for Mapping to Quality of Life and Capability Measures in Cataract Surgery Patients: From Cat-PROM5 to EQ-5D-3L, EQ-5D-5L, and ICECAP-O Using Mixture Modelling Click here for additional data file. Supplemental material, Appendix_2_online_supp for Mapping to Quality of Life and Capability Measures in Cataract Surgery Patients: From Cat-PROM5 to EQ-5D-3L, EQ-5D-5L, and ICECAP-O Using Mixture Modelling by Padraig Dixon, William Hollingworth and John Sparrow in MDM Policy & Practice
