# Supplementary material for: Mapping to Quality of Life and Capability Measures in Cataract Surgery Patients: From Cat-PROM5 to EQ-5D-3L, EQ-5D-5L, and ICECAP-O Using Mixture Modelling
Source: MDM Policy Pract. 2020 Apr 6;5(1):2381468320915447. doi: 10.1177/2381468320915447 (PMC7137115; doi:10.1177/2381468320915447)
Supplement: Appendix_1_online_supp – Supplemental material for Mapping to Quality of Life and Capability Measures in Cataract Surgery Patients: From Cat-PROM5 to EQ-5D-3L, EQ-5D-5L, and ICECAP-O Using Mixture Modelling [file Appendix_1_online_supp.doc]

This supplementary annex contains graphical outputs summarising conditional distribution outputs of observed versus predicted data at baseline and follow-up (Figures A1 and A2) and comparisons of observed versus predicted data by decile at baseline and follow-up (Figure A3 and A4).

**Figure A1 Comparison of actual and predicted follow-up distributions at baseline**

**Figure A2 Comparison of actual and predicted follow-up distributions at follow-up**

**Figure A3 Observed versus predicted data by decile at baseline**

**Figure A4 Observed versus predicted data by decile at follow-up**
